# Attention selectively enhances collision sensitivity in the superficial SC, PBGN and centromedial amygdala to looming stimuli in the upper visual field

**DOI:** 10.1162/IMAG.a.1432

**Published:** 2026-10-29

**Authors:** Jinyou Zou, Ye Wang, Chencan Qian, Peng Zhang

**Affiliations:** State Key Laboratory of Cognitive Science and Mental Health, Institute of Biophysics, Chinese Academy of Sciences, Beijing, China; Aier Academy of Ophthalmology, Central South University, Changsha, China; School of Stomatology and Ophthalmology, Xianning Medical College, Hubei University of Science and Technology, Xianning, China; University of Chinese Academy of Sciences, Beijing, China

**Keywords:** collision detection, attention, consciousness, subcortical pathway, high-resolution fMRI

## Abstract

Accurate detection of impending collisions is essential for survival. While multiple subcortical pathways have been implicated in the detection of looming stimuli, the roles of top-down attention and conscious awareness in this process remain unclear. Using high-resolution fMRI, we investigated how top-down attention and conscious awareness influence subcortical processing of approaching objects on collision versus near-miss trajectories. In healthy participants, the superficial layers of the superior colliculus (SCs), the parabigeminal nucleus (PBGN), and the centromedial amygdala (CMA) exhibited significantly stronger activation to hit-nasion than to near-miss trajectories in the upper visual field, but only when participants attended to the approaching object. Correlation and path analysis of collision sensitivity and beta-series functional connectivity consistently revealed a significant SC-PBGN connection. For stimuli presented in the blind visual field of hemianopic patients, significant functional connectivity was observed between the SC and both the pulvinar and the ventral tegmental area (VTA) in response to collision trajectories. Together, these findings suggest that attention selectively enhances collision sensitivity in the SCs-PBGN-CMA pathway for accurate detection of collision trajectory in the upper visual field, whereas the SC-pulvinar and SC-VTA pathways are associated with collision detection in the absence of conscious awareness.

## 1 Introduction

The ability to detect impending collisions is vital for our survival. Subcortical mechanisms for processing looming information have been found in both animals (Salay et al., 2018; Shang et al., 2015; Sun & Frost, 1998; Wang & Frost, 1992) and humans (Billington et al., 2011; Guo et al., 2024; Thieu et al., 2024). In rodents, recent studies have identified multiple subcortical circuits from the superior colliculus (SC) that are involved in innate defensive responses to looming stimuli, including SC-PBGN (parabigeminal nucleus)-Amygdala (Shang et al., 2015), SC-LP (lateral posterior nucleus of the thalamus, a pulvinar-like structure in rodents)-Amygdala (Wei et al., 2015), and SC-VTA (ventral tegmental areas)-Amygdala (Zhou et al., 2019). Optogenetic stimulation of the tectofugal pathways has been shown to trigger different types of defensive behaviors (Shang et al., 2018), depending on the internal and environmental context (Isa et al., 2020; Tseng et al., 2023). Despite significant advances in understanding the looming detection circuits in rodents, much less is known about the subcortical neural mechanisms of collision detection in humans.

The primate SC is a laminated structure located on the top of the brainstem. Its superficial layers primarily contain visuosensory neurons (May, 2006), whereas neurons in the intermediate and deep layers integrate saliency representations with top-down signals to construct a priority map that guides orienting behaviors (White, Berg, et al., 2017; White, Kan, et al., 2017; Zhaoping, 2016), such as attention and eye movements (Anderson & Rees, 2011; Krauzlis et al., 2013; Schneider & Kastner, 2009). Functional magnetic resonance imaging (fMRI) studies of the human SC have investigated its functional organizations of retinotopy (Benson et al., 2018; Katyal et al., 2010; Schneider & Kastner, 2005) and saccadic eye movements (Savjani et al., 2018). Using high-resolution fMRI approaches, previous studies have also investigated layer-specific visual responses (Loureiro et al., 2017; Wen et al., 2021; Zhang et al., 2015, 2016) and attentional modulations (Katyal & Ress, 2014; Katyal et al., 2010; Lovejoy & Krauzlis, 2017) in the human SC.

SC responses to looming stimuli have also been examined in fMRI studies. BOLD fMRI activity in the SC is more sensitive to looming than to receding stimuli, both when subjects attended to the stimuli in a time-to-collision (TTC) judgment task (Billington et al., 2011) and during passive viewing of approaching threatening object (Clery et al., 2017; Thieu et al., 2024, 2026). These studies suggest that the SC plays important roles in multiple cortical–subcortical networks for detecting looming threats. Our recent work showed that the SC exhibits sensitivity to collision compared to near-miss trajectories even without attention and awareness to the looming stimuli (Guo et al., 2024). This effect was observed when healthy participants were doing color detection task at fixation and when the looming stimuli were presented in the blind visual field of hemianopic patients with unilateral lesions to the geniculostriate pathway. However, while the SC activity was highly sensitive to attentional modulation as suggested by many previous studies (Hafed et al., 2023; Katyal & Ress, 2014; Katyal et al., 2010; Lovejoy & Krauzlis, 2017; White, Berg, et al., 2017; White, Kan, et al., 2017), we found no significant collision-sensitive activity when healthy participants attended to the looming stimuli in a trajectory discrimination task. We speculate that this lack of collision sensitivity may be attributable to response saturation in the SC at a short TTC (33 ms) under attended condition. Thus, how top-down cognitive factors, such as attention and awareness, modulate neural activity to looming stimuli in the SC and its downstream subcortical nuclei remains unclear.

In this study, we aim to investigate the role of top-down attention and conscious awareness in subcortical collision detection in human observers using high-resolution fMRI approaches. To avoid response saturation observed in the previous study, we employed looming stimuli with a longer TTC in the fMRI experiment (138 ms vs. 33 ms in Guo et al. (2024)). In separate sessions, participants either attended to and judged the trajectory of the looming object (attended condition), or performed a demanding central fixation task while ignoring the looming stimuli (unattended condition). Visual stimuli were identical in both conditions, with the only difference being the focus of attention: either on the looming stimuli or on the central fixation (Bahrami et al., 2007; O’Connor et al., 2002). The results revealed enhanced responses to hit-nasion versus near-miss trajectories in the upper visual field in the superficial SC, PBGN, and centromedial amygdala (CMA) in the attended condition, but not in the unattended condition. Path analysis of collision sensitivity further revealed significant SC–PBGN connectivity, specific to stimuli in the upper visual field. We also reanalyzed data from hemianopic patients in our previous study and found significant SC-pulvinar and SC-VTA connectivity to looming stimuli on collision trajectories presented in their blind visual field.

## 2 Methods

### 2.1 Participants

A total of 23 healthy young adults (9 males, 21 naive participants) participated. This sample size provides 80% power (Type I error < 0.05) for detecting an effect size (Cohen’s d) greater than 0.611 for the key comparison between hit-nasion and near-miss trajectories in the current study. The age of the participants ranged from 20 to 40 years old, with normal or corrected-to-normal vision. All participants provided written informed consent before the experiments. All procedures performed in studies involving human participants were in accordance with the ethical standards of the Institutional Review Board of the Institute of Biophysics (Ethic No. 2012-IBP-011) and with the 1964 Helsinki declaration and its later amendments or comparable ethical standards. The data of 12 hemianopic patients with unilateral lesions of the geniculostriate pathways from our previous study (Guo et al., 2024) were re-analyzed for the path analysis. Patients lost their conscious vision in one-half or a quadrant of the visual field (relative sensitivity less than -20 dB and p < 0.5% compared with normal population in both eyes), while they had normal or corrected-to-normal vision outside the blind visual field. For these patients, the clinical characteristics, stimuli and procedure, and MRI data acquisition and preprocessing, were described in our previous study.

### 2.2 Apparatus, stimuli, and procedure

Visual stimuli were generated using MATLAB with Psychtoolbox 3.0 (Brainard, 1997; Pelli, 1997). 3D spheres, rendered from slightly different perspectives for each eye, were presented using an MRI-compatible polarized LCD display (3D BOLD screen 24, Cambridge Research Systems). Participants viewed the screen via a mirror mounted inside the head coil, at a viewing distance of 130 cm (Supplementary Fig. S1). As shown in Supplementary Figure S2, the sphere appeared at a virtual distance of 11.3 m and vanished at 3.3 m from the observer, moving at a speed of 24 m/s. The time-to-collision (TTC) at the final frame was 138 ms. The trajectory began in one of the four visual field quadrants, with a horizontal offset of 0.65 m and a vertical offset of 0.38 m. The sphere would either hit the nasion (hit-nasion), hit the ipsilateral eye (hit-eye; 3 cm from nasion), or slightly miss the head (near-miss) (Fig. 1A, 1B). The near-miss trajectory was adjusted for each individual to achieve approximately 50% “hit” responses in a training session outside the scanner, resulting in an average impact point of 5.9 cm from the nasion. To match their retinotopic locations, the on-screen projections of the looming stimuli were aligned to the center of mass. The on-screen image expanded from 0.3° to 1.0° of visual angle over 330 ms, with a mean eccentricity of 3.8°. For stereoscopic stimulus presentation, the interocular distance was set to 6 cm according to the population-average inter-pupillary distance (IPD). Then, we generated the stimuli and estimated the impact point (0, 3, and 5.9 cm for hit nasion, hit eye, and near miss conditions) based on this setting. During the experiment, we measured the actual IPD of each participant, which had a mean of 6.1 cm and a range of 5.5–6.6 cm. The maximum horizontal retinotopic position error of the stimulus introduced by IPD variation across participants was 0.016°, which is unlikely to affect the experimental results. Luminance polarity was counterbalanced across participants: dark stimuli (mean luminance = 2.9 cd/m²) were presented on a bright background (5.8 cd/m²) for 11 participants (first group), while bright stimuli (mean luminance = 5 cd/m²) were presented on a black background (~0 cd/m²) for the remaining 12 participants (second group).

**Fig. 1.**
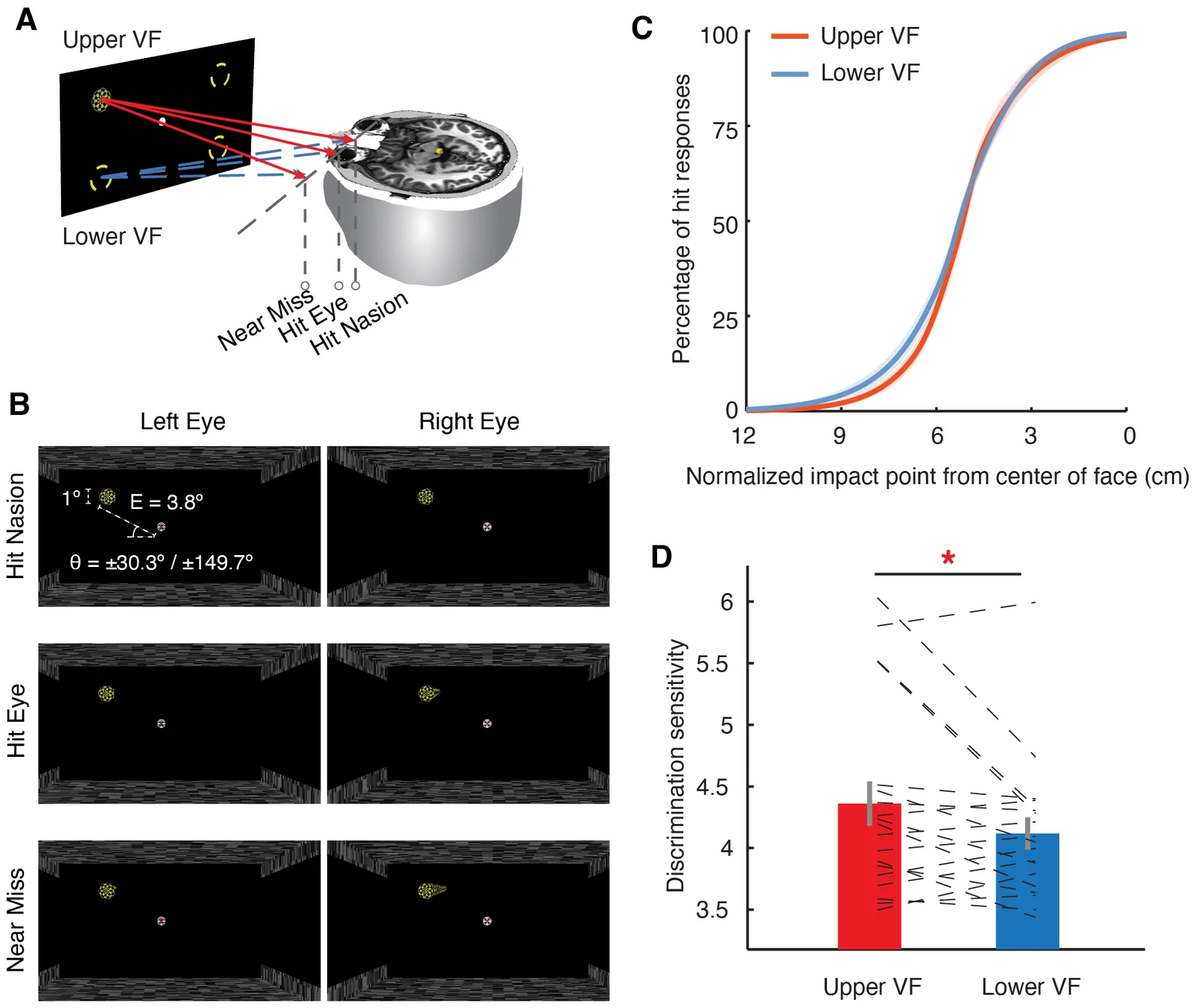
Schematic diagrams of looming stimuli and behavioral performance. (A) Visual stimuli depicting an approaching object were presented using a MRI-compatible 3D monitor (monitor with polarized filters for 3D perception) and polarized glasses (Supplementary Fig. S1). The trajectory would either be a direct hit (hit nasion, hit eye) or a near miss of the observer’s head. (B) The images presented to the left and right eyes were shown for three trajectory conditions. All video frames were superimposed to illustrate the motion trajectory. E and θ represent the eccentricity and polar angle, respectively, of the retinotopic stimulus location. This example illustrates looming stimuli in the upper-left visual field. In the actual experiment, stimuli could appear at symmetrical positions in all four quadrants of the visual field (θ = ± 30.3º / ±149.7º). (C) The percentage of hit responses against the extrapolated impact point was fitted with a normal cumulative distribution function. (D) Discrimination sensitivity was defined as ln(1/σ), where σ is the standard deviation of the fitted response function. The shaded areas and error bars indicate the standard error of the mean. VF: visual field. *p < 0.05.

The scanning protocol differed slightly between the two groups of healthy participants. In the first group, attended and unattended runs of the looming experiment were collected separately in two sessions (12 runs per session or attention condition). Each run consisted of 12 trials (3 trajectories × 4 quadrants, 242 s). A total of 144 trials (4 quadrants × 3 trajectories × 12 repetitions) were collected per attention condition. Trials were presented in a random order and inter-stimulus intervals (ISIs) of 10, 12, or 14 s, with null events randomly added to improve the estimation efficiency of GLM. In attended runs, participants were instructed to keep central fixation and judged whether the looming object was on a collision course. In unattended runs, they performed an attention demanding rapid serial visual presentation (RSVP) task at fixation, detecting letter targets (white ‘T’ and blue ‘O’) among distractors (letter size: 0.35°; duration: 150 ms; rate: 2 letters/s). For the second group of healthy participants, each session included 4 attended runs and 4 unattended runs (18 trials and 288 s for each run). Run order was counterbalanced both within and between sessions. Trial sequence was first randomly shuffled across all runs and assigned into each run. RSVP letters were presented at fixation in both attention conditions. Other experimental details were identical as the first group.

Four runs of retinotopic localizer were acquired for every participant in a third session. Each run comprised 16 cycles, each consisting of an 8-s stimulus block followed by an 8-s fixation block (256 s per run). During each stimulus block, a checkerboard disc flicker (1.9° diameter, reversing contrast at 7.5 Hz) was presented for 500 ms in alternation with 500-ms fixation period. Stimuli were presented at the same locations as in the main experiment. For each quadrant location, 4 stimulus blocks were presented per run, yielding 16 blocks per location across the 4 runs. Participants passively viewed the stimuli while maintaining central fixation.

For hemianopic patients (Refer to Guo et al. (2024) for details), similar looming stimuli were presented on a 2D screen. In different trials, a sphere moved along hit (hit-eye), near-miss, or receding (backward-moving) trajectories. The time-to-collision (TTC) at the final frame was 115 ms, corresponding to a distance of 2.75 m from the observer. Stimuli were presented in either the blind or the intact visual field of each patient. During fMRI scans, patients performed a central fixation task that was irrelevant to the moving sphere. Subcortical fMRI responses to stimuli in the blind visual field (hit and near-miss conditions) were used for path analysis and beta-series connectivity analysis in the current study.

### 2.3 MRI data acquisition and preprocessing

MRI data were acquired using a 3T MRI scanner (Siemens Prisma Fit, Erlangen, Germany) and a 20-channel head coil (Nova Medical, Cambridge, MA, USA) at Beijing MRI center for Brain Research. Functional images were acquired with a gradient recalled echo planar imaging (GE-EPI) sequence (2-mm isotropic voxels, 54 axial slices, 192-mm FOV, 96 × 96 matrix, TR/TE = 2000/31.4 ms, flip angle = 80°, multiband factor = 2, pixel bandwidth = 2170 Hz, echo spacing = 0.46 ms, no in-plane acceleration/GRAPPA, no partial Fourier). High-resolution anatomic images were obtained with a T1w MPRAGE sequence (1-mm isotropic voxels, 192 sagittal slices, 256 × 256 matrix, TR/TE = 2600/3.02 ms, flip angle = 8°). Pulse and respiration data were recorded for the first 11 participants.

MRI data were pre-processed using AFNI (Cox, 1996) and Freesurfer (Dale et al., 1999; Fischl et al., 1999). The pre-processing steps included: RETROICOR physiological noise removal (Jo et al., 2010); slice timing correction; motion correction and co-registration of functional images to T1w anatomical volumes; functional data resampled (cubic interpolation) at 1-mm isotropic resolution; scaled to percent signal change. The upsampling procedure in preprocessing can help achieve better effective fMRI resolution (Wang et al., 2022), and has been used in our previous studies to investigate layer-specific activity in subcortical nuclei (Wen et al., 2021; Zhang et al., 2015, 2016).

In volume-based analysis, the anatomical volume was first transformed to Talairach space (TT_N27 in AFNI) using affine transformation followed by a nonlinear warping procedure (AFNI 3dQWarp). A spherical mask was applied to the subcortical regions to constrain the registration and improve alignment quality. The transformation parameters were then applied to the functional volumes (AFNI 3dNwarpApply). Results were visually inspected for each participant. Supplementary Figure S3 shows the registration quality. For the retinotopic localizer, 2-mm FWHM smoothing was performed within the subcortical ROIs (AFNI 3dBlurinMask) to enhance SNR for localization.

In surface-based analysis, T1-weighted anatomical images were segmented into gray matter, white matter, and cerebral spinal fluids using FreeSurfer’s recon-all option. The functional volumes after motion correction and co-registration were mapped onto the cortical surface. Spatial smoothing was performed on the cortical surface with a 6-mm FWHM gaussian kernel. Surface data were registered to a standard surface (std.141 in AFNI) using a standard-mesh method via sulcal-gyral pattern matching (Argall et al., 2006).

After pre-processing, a general linear model (GLM) with a canonical HRF (BLOCK4(0.5,1) in AFNI) was used to estimate the BOLD response (beta value) for each stimulus condition. Baseline regressors included zero-, first-, and second-order polynomials. Noise regressors included 6 rigid motion parameters, and a time course from the anterior cerebellum to reduce physiological noise in subcortical regions, especially the SC (Schneider & Kastner, 2005; Wall et al., 2009). For accurate estimation of collision-sensitive response in the SC, a faster HRF with an early time to peak (4 s), early fall time and moderate undershoot fraction (WAV(0,1,3,4.2,0.5,2.8) in AFNI) was used in the GLM (DeSimone et al., 2015; Wall et al., 2009). Event-related average of response time course in the SC was calculated using the detrended time series (AFNI 3dTproject), after removing baseline and noise regressors. The signal intensity at stimulus onset and 2 s before were used as the baseline.

During the analysis of looming responses, trials for the left and right visual hemifields were combined. For retinotopic regions, including the SC, LGN, and pulvinar, their responses to contralateral looming stimuli were analyzed: left-hemisphere ROI responses to the right hemifield stimuli and right-hemisphere ROI responses to the left hemifield stimuli. While, for other structures, including PBGN and VTA, responses in bilateral ROIs were averaged.

### 2.4 ROI definition

For brain areas with clear retinotopic organization, we used independent retinotopic localizers within anatomical constraints to define ROIs. For other regions, ROIs were defined directly using structural atlases or anatomical landmarks. Due to the functional complexity and relatively large volume of the amygdala, functional connectivity with the SC was used to further constrain the ROI selection (see below for details).

The whole anatomical ROIs of the SC were manually drawn on T1w anatomical images. The depth of each voxel was defined as the minimum distance to the surface of the SC (Fig. 2A). While a Euclidean distance method was used for depth estimation, layer definition approaches that account for surface curvature and non-uniform thickness may provide more accurate depth estimates (Katyal et al., 2010; Truong et al., 2020). Voxels of 0 to 1.5 mm depth were defined as the superficial layers, and those of 1.5 to 3 mm depth as the intermediate layers. For the laminar analysis, the anatomical template and functional images were upsampled to 0.5-mm isotropic resolution. Retinotopic ROIs of the superficial SC were defined as the most significant 15 mm³ of voxels (p < 0.01 for all voxels) with contralateral-ipsilateral activations, separately for the UVF and LVF. This ROI volume was chosen to create separated ROIs for the UVF and LVF. It constitutes 8.5% of the total superficial layer volume on one side of the SC and is roughly aligned with previous findings (Katyal et al., 2010). Different volumes of the selected ROIs led to qualitatively similar conclusions for the subsequent analysis (Supplementary Fig. S4). All voxels in the intermediate layers were included as the ROI for the intermediate SC (91 mm³ for one side). To control for the difference in ROI selection, we also defined retinotopic ROIs in the intermediate layer using the same method as for the superficial layer, even though retinotopic activation did not reach significance in the intermediate layer—likely due to reduced response amplitude or the limited fMRI resolution for deeper layers. In the ATT-UVF condition, the results showed no trajectory effect (F(2, 42) = 0.29, p = 0.749; hit-nasion vs. near-miss: p = 0.59). LGN and pulvinar ROIs were defined by a combination of anatomical constraint and significant localizer activation (voxel p < 0.02, the threshold was selected to ensure the cluster size > 10 mm^3^) (Arcaro et al., 2015; DeSimone et al., 2015). Retinotopic ROIs were obtained separately in the lateral and inferior pulvinars. These ROIs were combined for subsequent analyses, as no collision sensitivities were found in either ROI. The ROIs for the PBGN and VTA were defined by anatomical landmarks. PBGNs are located bilaterally on the middle section of the brain stem, below the inferior colliculus and above the substantia nigra. We carefully studied the location and anatomy of the PBGN according to human brain atlas from Nissl stained images (Mai et al., 2016; Pzxinos et al., 2012), and the ROIs were defined on T1w-MPRAGE volumes as a cylinder in-between the inferior colliculus (IC) and the substantia nigra (SN): the lateral and posterior boundaries were at the surface of the brain stem, the medial boundary was 4 mm from the lateral boundary, the superior boundary was at the bottom of the IC, the inferior boundary was at the top of the SN, and the anterior boundary was at the anterior end of the IC. The volume of the final ROI was 120 mm^3^ and 115 mm^3^ for the left and right PBGN. VTA ROIs were manually defined from the midbrain on T1w anatomical images refer to a previous study (Ballard et al., 2011).

**Fig. 2.**
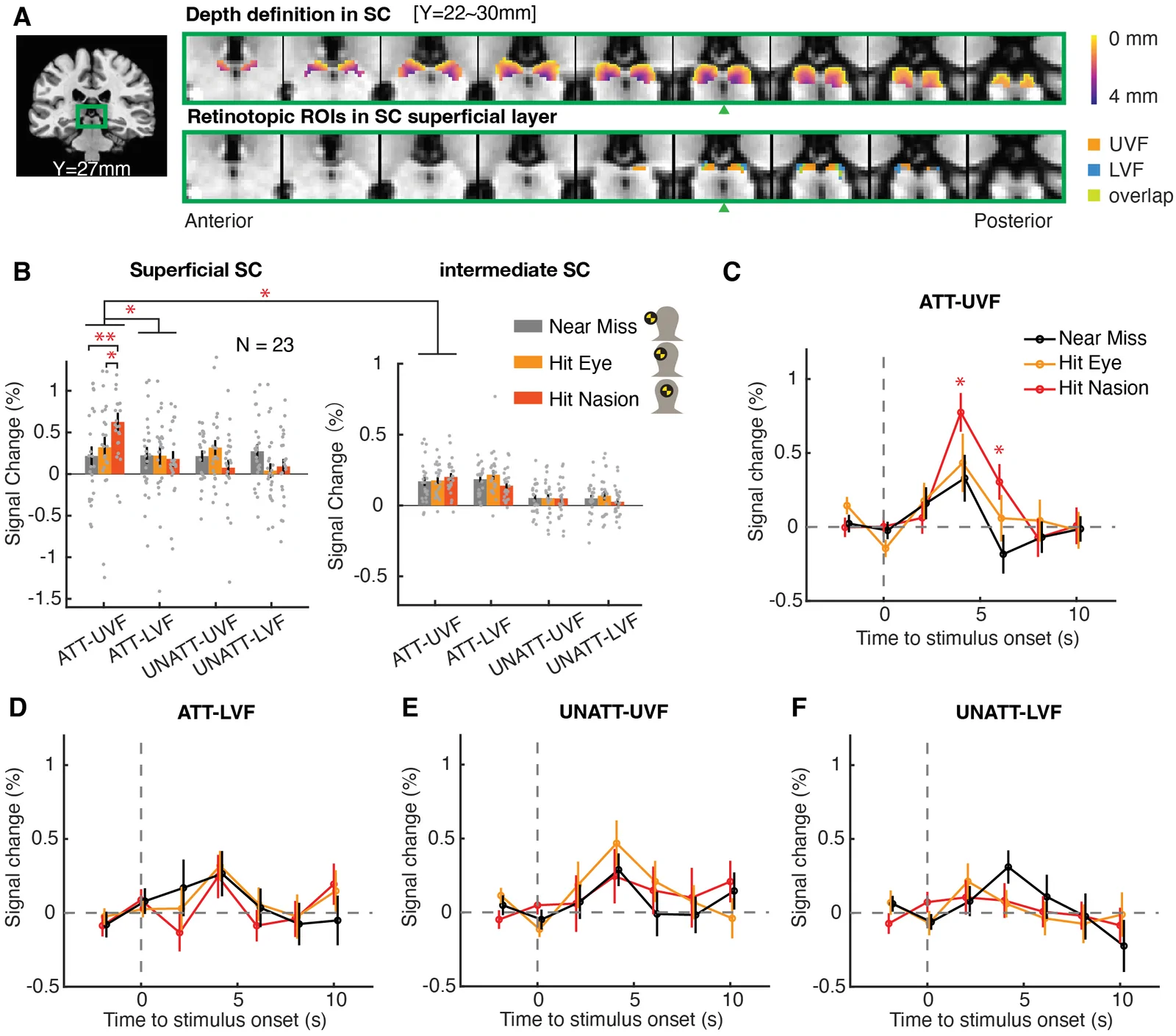
Layer-dependent ROIs and fMRI responses of the superior colliculus (SC). (A) The upper and lower rows illustrate the depth map in the SC and the retinotopic ROIs (contralateral vs. ipsilateral, voxel p < 0.01 across subjects) in the superficial layers, respectively. Coronal slices were presented from anterior to posterior at a 1-mm step. The enlarged slice corresponding to the full image in the left panel is marked by a triangle below. Coordinates are in AFNI TLRC space (positive Y = posterior). The images shown here are ROIs or group-average functional maps on a standard template (TT_N27). (B) BOLD responses of the superficial (left panel) and intermediate (right panel) SC to looming stimuli at different trajectories. Gray dots denote the data of individual participants. (C-F) BOLD time series in the superficial SC to the looming stimuli. *p < 0.05 (for hit-nasion vs. near-miss conditions in (C)), **p < 0.01. Error bars indicate standard errors of the mean (S.E.M.). The results were obtained from the responses of bilateral ROIs to contralateral stimuli. ATT, attended; UNATT, unattended; UVF, upper visual filed; LVF, lower visual field.

The amygdala complex was divided into a basolateral (BLA) and a centromedial (CMA) part based on the probabilistic cytoarchitectonic atlas derived from human post-mortem studies (Amunts et al., 2005; Eickhoff et al., 2005). Given that the neural circuits from the SC to amygdala have been shown mediating defensive behaviors to looming stimuli (Koller et al., 2019; Pegna et al., 2005; Rafal et al., 2015; Shang et al., 2015; Wei et al., 2015), BLA and CMA ROIs were defined as voxels showing significant functional connectivity with the SC using a beta-series method (refer to Section 2.6) (Rissman et al., 2004). In which, the response for each trial was estimated with a separate regressor in a GLM, generating a series of beta values. Beta values were Z-scored within each condition. A linear regression was conducted with the beta series from the target (SC) and source (BLA/CMA) ROIs, and the regression coefficient was obtained as the strength of functional connectivity. A leave-one-subject-out (LOSO) procedure was used to define the ROIs for each participant. BLA and CMA ROIs were defined as the largest significant cluster in the nucleus from the group-level connectivity map from all other participants (voxel p < 0.02, cluster size > 10 mm³).

Frontoparietal ROIs were defined on the standard surface (std.141 in AFNI) using HCP-MMP1 atlas (Glasser et al., 2016). ROIs for visual cortices were defined by significant activations in the retinotopic localizer (p < 0.05 after FDR correction). V1–V3 ROIs were defined by significant activations in the retinotopic localizer separately for each quadrant (quadrant stimulus vs. fixation). hMT+ was defined by the lateralization effect (Contralateral vs. Ipsilateral, UVF and LVF ROIs were combined due to the less clear separation). Due to the lack of clear retinotopic organization, VO was defined by the mean activation of all localizer stimuli vs. baseline. For V1–V3, and hMT+ with clear retinotopic organizations, responses to looming stimuli in contralateral visual field were used for ROI-based analysis. For other cortical areas with weak or no retinotopic organization, ROI responses to contralateral and ipsilateral stimuli were averaged. For all brain areas, ROI responses in the left and right hemispheres were averaged.

For hemianopic patients, in the absence of a retinotopic localizer, a leave-one-run-out (LORO) procedure was used to define the SC and pulvinar ROIs for the connectivity analysis. Specifically, within the anatomical ROIs, voxels most responsive to the collision trajectory (16–17.28 mm³, depending on fMRI resolution, hit-miss p < 0.05 for all voxels) in N-1 runs were used to calculate the ROI-averaged response for the left-out run. The LORO procedure was repeated for each run and the results were averaged across all runs. Since collision-sensitive activation was mainly observed in the ventromedial pulvinar, this sub-region was used as the anatomical ROI for the pulvinar in the LORO analysis. ROI definitions for the PBGN, VTA and CMA were the same as those used for healthy participants.

### 2.5 Path analysis

Path analysis with SEM methods was performed to estimate the effective connectivity between looming-sensitive ROIs, including SC, PBGN, pulvinar, VTA, and CMA, based on the across-subject correlations of collision sensitive responses (CSRs) among brain regions. We first computed the ROI-averaged CSRs for each participant in each attended/unattended × UVF/LVF condition, and Z-scored across participants for each ROI and condition. Then, the across-subject correlations of normalized CSRs among brain regions were used to estimate an SEM model with fixed connections (Fig. 5B) based on prior anatomical evidence (May, 2006; Rafal et al., 2015; Shang et al., 2015; Wei et al., 2015; Zhou et al., 2019; Zubair et al., 2021). The path coefficients were fitted by maximum likelihood estimate in the SEM with Lavaan software 0.6–17 (Merkle & Rosseel, 2018; Rosseel, 2012) in R. The model was fitted separately for each of the four conditions (ATT-UVF, ATT-LVF, UNATT-UVF, UNATT-LVF) in healthy participants and for stimuli presented in the blind visual field of hemianopic patients (unaware condition). Finally, the path coefficients were compared between different conditions using two-sample Z tests:

$$Z=\frac{\beta_{1}-\beta_{2}}{\sqrt{SE_{1}^{2}+SE_{2}^{2}}}$$

in which β and SE denote the strength and standard error of the estimated connection.

### 2.6 Functional connectivity

To measure functional connectivity between the SC and its downstream subcortical regions (PBGN, pulvinar, and VTA), we adopted a beta-series method (Rissman et al., 2004). For each ROI, we first extracted the mean time series averaged across all voxels. This ROI-averaged time series was then used to estimate single-trial responses via a GLM with a separate regressor for each trial, yielding a series of beta values for each collision trajectory (hit-nasion, hit-eye) × visual field × attention condition. The beta series for each condition were then Z-scored. For each visual field × attention condition, the hit-nasion and hit-eye beta series were concatenated. Functional connectivity was then computed as the Pearson’s correlation between the concatenated beta-series of each pair of ROIs.

### 2.7 Individual-level sensitivity analysis

To quantify collision sensitivity at the individual level, we extracted single-trial beta estimates from the superficial SC ROI (without Z-score). For each participant, we computed d’ and AUC using the trial data to distinguish hit-nasion from near-miss trajectories. We calculated d’ as follows:

$$d^{\prime }=\frac{\mu_{HitNasion}-\mu_{NearMiss}}{\sqrt{\frac{\sigma_{HitNasion}^{2}+\sigma_{NearMiss}^{2}}{2}}}$$

where μ and σ are the mean and standard deviation, respectively, of the trial-wise beta values for each condition. We computed AUC using MATLAB Perfcurve function, setting hit-nasion trials as the positive class and near-miss trials as the negative class.

### 2.8 Behavioral performance

We analyzed the behavioral responses in the attended conditions using the same methods as in our previous study (Guo et al., 2024). Specifically, the percentage of hit responses as a function of extrapolated impact point from nasion (corresponding to different trajectories) was fitted for each participant using cumulative normal distributions with two parameters: μ and σ. The fitting was performed for the UVF and LVF separately. Discrimination sensitivity was obtained as ln(1/σ). Higher values indicate steeper psychometric function and better discrimination. Finally, the discrimination sensitivity was compared between the UVF and LVF using paired t-test. As the number of trials was limited in the fMRI experiment, we excluded four subjects with response rates lower than 85% from this analysis to avoid outliers caused by an insufficient number of effective trials in some individuals’ data.

Additionally, all participants completed a brief training session prior to the fMRI experiment to learn the task. Each subject received specific instructions on maintaining fixation. Although we did not monitor eye movements during scanning, 17 subjects participated in another behavioral session with eye tracking. The other subjects could not participate in the eye-tracking experiment for personal reasons.

### 2.9 Statistics

We performed LME analysis for the ROI-averaged responses separately for the attended and unattended conditions. Trajectory (hit nasion, hit eye, or near miss), visual field (UVF or LVF), and stimulus luminance (bright or dark; between-subject factor) were included as fixed effects, with participant as a random grouping factor. Collision sensitivity was first assessed as an interaction effect between trajectory and visual field or a main effect of trajectory. Then, the trajectory effects in the UVF and LVF were tested in separate LME models. Finally, post hoc paired t-tests with Holm corrections were performed between different trajectory conditions if a significant effect of trajectory was observed.

Whole-brain analysis was performed on the cortical surface to detect potential collision sensitivity without making specific prior assumptions on the ROIs. At a threshold of p < 0.001, group-level activations were corrected for family-wise error (p < 0.05) using a cluster size of 140 mm^2^. The cluster-size threshold was obtained using a simulation procedure with self-generated all-noise data on the same surface model and blurring level (AFNI slow_surf_clustsim.py, iteration = 10000000).

Beta series functional connectivity was first obtained for each individual. Then, the significance of connectivity was examined with non-parametric exact permutation test considering the skewed distribution of connectivity data (same as correlation coefficient). The p values were further corrected for multiple comparison (6 comparisons) problems with Holm method. For each subject group (healthy participants in ATT-UVF conditions, patients in unaware conditions), we also compared the connectivity between different pathways using LME models with subject as a random effect. Fisher transformation was applied to the connectivity data before the LME analysis. The following paired tests were performed using exact permutation test with Holm correction if a significant effect of pathways were observed. Finally, we compared the connectivity for the same pathways between the two subject groups using permutation test (permutation=10^7^) for independent samples with Holm correction (3 comparisons).

## 3 Results

An approaching sphere was presented stereoscopically using a polarized 3D display and 3D glasses (Fig. 1A and Supplementary Fig. S1). The sphere moved through a virtual tunnel towards the observer at a constant speed of 24 m/s. The sphere traveled from 11.3 m to 3.3 m in front of the observer (Supplementary Fig. S2). The TTC of the last frame was 138 ms, much longer compared to the previous study (33 ms), in order to avoid response saturation in subcortical regions. The trajectory would either hit the nasion (hit nasion), hit the ipsilateral eye (hit eye), or just miss the head (near miss) of participants (Fig. 1A and 1B). In the attended condition, participants responded whether the approaching object would hit or miss their heads. In the unattended condition, they performed a demanding rapid serial visual presentation (RSVP) task at fixation and ignored the looming stimuli. To control for the effects of luminance-induced pupil dilation on subcortical responses, bright stimuli were presented on a black background in 11 participants, while dark stimuli were presented on a light gray background in another 12 participants. Checkerboard flickers in the same size and location as the looming stimuli were employed to localize retinotopic regions of interest (ROIs). In the attended conditions, participants could precisely discriminate the trajectories of looming objects, with higher behavioral sensitivity in the upper visual field (UVF) than in the lower visual field (LVF) (Fig. 1C and 1D; t(18) = 2.10, p < 0.05, d = 0.482). This replicates the behavioral results in our previous study (Guo et al., 2024).

### 3.1 Attention enhanced activity in the superficial SC, PBGN, and CMA to hit-nasion versus near-miss trajectories in the upper visual field

We first analyzed fMRI responses in the superior colliculus (SC). Given that neurons in the superficial and intermediate layers may be differently involved in processing looming stimuli (Shang et al., 2015; Wei et al., 2015), we analyzed layer-dependent responses in the SC. Based on the laminar organization of the human SC (Tardif & Clarke, 2002), voxels within 1.5 mm below the SC surface were defined as the superficial layers, while those between 1.5 mm and 3 mm in depth were classified as the intermediate layers. In the retinotopic localizer, counterphase flickering checkerboard in the upper and lower visual field activated the medial and lateral portions of the superficial SC, respectively (Fig. 2A lower row). This finding is consistent the retinotopic organization of the human SC suggested by previous fMRI studies (Benson et al., 2018; Katyal et al., 2010; Schneider & Kastner, 2005), and consistent with the neuroscience evidence that neurons in the superficial SC mainly process visuosensory information (May, 2006; Zhang et al., 2015, 2016). Thus, for the superficial SC, we selected voxels within the superficial layers that were significantly activated by the retinotopic localizer as the ROI. Separate ROIs were defined for the UVF and LVF (Fig. 2A). All voxels within the intermediate layers were included as the ROI for the intermediate SC. Studies on rodents have shown that tectofugal pathways primarily mediate defensive responses to looming stimuli in the UVF (Deichler et al., 2020; Isa et al., 2020; Wei et al., 2015; Zhou et al., 2019). This UVF bias is consistent with our human behavioral data. Additionally, a vertical asymmetry across the visual field has been observed in primates in many behavioral tasks and responses in cortical and subcortical areas (Hafed & Chen, 2016; Himmelberg et al., 2022, 2023). Therefore, in the following analysis, we examined the neural responses to stimuli in the UVF and LVF separately to investigate potential visual field bias.

A linear mixed effects (LME) analysis was conducted on ROI-averaged responses, separately for the attended (ATT) and unattended (UNATT) conditions. Trajectory (hit nasion / hit eye / near miss) and visual field (UVF / LVF) were included as fixed effects, with participant as a random grouping factor. In the superficial SC (Fig. 2B, left), we observed a significant interaction between trajectory and visual field in the attended condition (F(2, 84) = 3.54, p = 0.034). Follow-up analysis revealed a significant effect of trajectory in the UVF (F(2, 42) = 5.43, p = 0.008; hit-nasion vs. near-miss: t(22) = 3.18, p = 0.008, d = 0.756; hit-nasion vs. hit-eye: t(22) = 2.33, p = 0.049, d = 0.554), but not in the LVF (main effect of trajectory: p = 0.932). Stimulus luminance did not yield any significant effect (all p > 0.1) when it was included in the LME model, indicating that the observed collision sensitivity in the superficial SC was not attributable to changes in stimulus luminance. Furthermore, collision sensitivity (hit-nasion vs. near-miss) was robust across different voxel inclusion thresholds for the retinotopic ROIs (Supplementary Fig. S4). Event-related average of BOLD time series (Fig. 2C) further revealed significantly greater responses to hit-nasion compared to near-miss trajectories in the ATT-UVF condition (peak response at 4 s from stimulus onset: t(22) = 2.25, p = 0.035, d = 0.469; see Fig. 2D–2F for other conditions). In the unattended condition, however, there were no significant main effects of trajectory (p = 0.158), visual field (p = 0.356), or their interaction (p = 0.11).

To further demonstrate the effect of attention on collision sensitivity, we compared the SC responses to looming trajectories in attended and unattended conditions within the same LME model. Since stimuli in the lower visual field produced weak SC responses and no collision sensitivity in any condition, we focused the analysis in the upper visual field. Results showed a significant attention*trajectory interaction (F(2, 110) = 5.26, p = 0.007), suggesting that top-down attention modulates collision-sensitive responses in the superficial SC.

In contrast to the collision sensitive responses observed in the superficial SC, the intermediate SC showed no significant effect of trajectory or trajectory-related interactions (all p > 0.1; Fig. 2B, right). The layer-specific effect on collision sensitivity was supported by a significant trajectory*layer interaction (F(2, 110) = 3.19, p = 0.045). Moreover, the response difference between hit-nasion and near-miss conditions was significantly larger in the superficial layers compared to the intermediate layers of the SC (t(22) = 2.85, p = 0.009, d = 0.59). To evaluate the spatial specificity of fMRI signal in the SC, we estimated the FWHM of SC responses using AFNI’s 3dFWHMx on the residual time series after GLM. The mean FWHM across participants was 2.41 mm (range: 2.12–2.69 mm).

To quantify collision sensitivity at the level of individual, we extracted single-trial beta values from the superficial SC and computed d’ and the area under the ROC curve (AUC) for discriminating hit-nasion from near-miss trajectories in each participant. In the ATT-UVF condition, d’ was significantly greater than zero (group mean = 0.27 ± 0.36, 95% CI = 0.12~0.43; t(22) = 3.63, p = 0.001, d = 0.76), and AUC was significantly above the chance level of 0.5 (group mean = 0.58 ± 0.09, 95% CI = 0.54~0.62; t(22) = 4.11, p < 0.001, d = 0.86). In contrast, no significant collision sensitivity was observed in other conditions (all p > 0.1). This individual-level analysis further confirms the collision sensitivity observed in group-averaged fMRI responses.

The downstream subcortical targets of the SC include the PBGN (Shang et al., 2015), the pulvinar (Wei et al., 2015), and the VTA (Zhou et al., 2019). ROIs for the PBGN and VTA were defined using anatomical landmarks on the T1-weighted structural images (Ding et al., 2016; Mai et al., 2016), whereas the pulvinar ROI was delineated based on retinotopic activations and a retinotopic atlas (Arcaro et al., 2015) (Fig. 3A) . In the attended condition, LME analysis of ROI-averaged responses (Fig. 3B) revealed a significant trajectory × visual field interaction in the PBGN (F(2, 84) = 3.91, p = 0.024). Follow-up analysis revealed a significant effect of trajectory in the UVF (F(2, 42) = 5.92, p = 0.005; hit nasion vs. near miss: t(22) = 3.12, p = 0.01, d = 0.685; hit nasion vs. hit eye: t(22) = 2.81, p = 0.015, d = 0.618), but not in the LVF (main effect of trajectory: p = 0.761). No significant effect of trajectory or related interactions was observed in the pulvinar or the VTA (all p > 0.38). In the unattended condition, no significant main effects of trajectory or trajectory × visual field interactions were found in any of the ROIs (all p > 0.13; Fig. 3C).

**Fig. 3.**
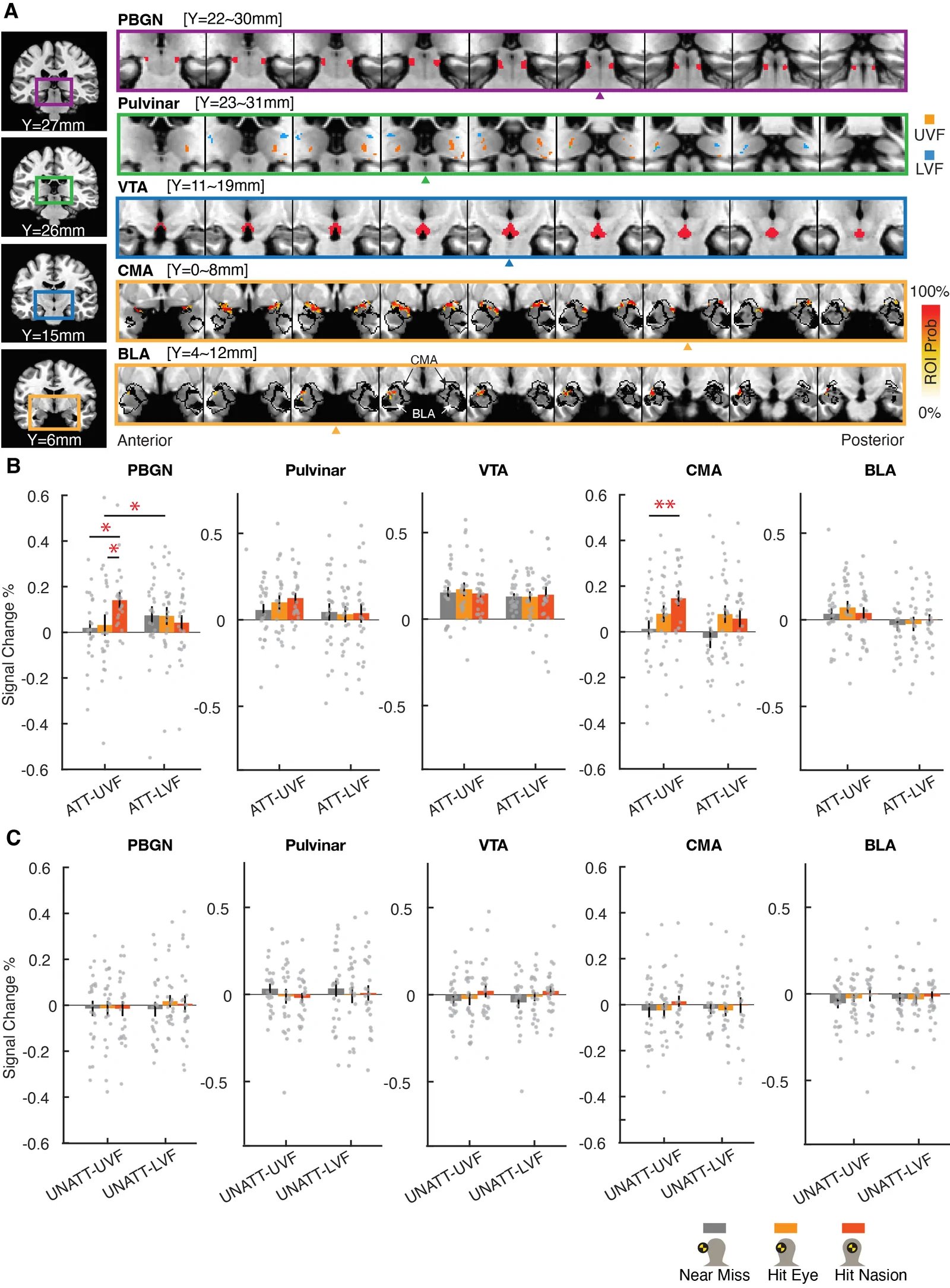
Looming responses in the downstream subcortical nuclei of the SC. (A) ROIs of the nuclei in coronal slices. The pulvinar ROIs were defined by independent retinotopic localizers, separately for the UVF and LVF (Contralateral vs. Ipsilateral, voxel p < 0.02 across subjects). CMA and BLA ROIs were defined as voxels with significant functional connectivity with the SC. Voxel color indicates the probability of ROI overlap (ROI Prob) across participants. The black lines indicate the anatomical boundaries for subnuclei of the amygdala. The enlarged slice corresponding to the full image in the left panel is marked by triangles below. Y indicates Talairach coordinates (positive Y = posterior). The images shown here are ROIs or group-average functional maps on a standard template (TT_N27). (B, C) ROI-averaged BOLD responses to looming stimuli in the attended (B) and the unattended (C) conditions. PBGN, parabigeminal nucleus; VTA, ventral tegmental areas; CMA, centromedial complex of amygdala; BLA, basolateral complex of amygdala. *p < 0.05, **p < 0.01. Error bars indicate S.E.M. For the pulvinar, responses to contralateral stimuli were analyzed. For other non-retinotopic ROIs, responses to both hemifields were averaged. For all brain regions, results from ROIs in both hemispheres were combined. ATT, attended; UNATT, unattended; UVF, upper visual filed; LVF, lower visual field.

Finally, we examined the amygdala, a key terminal of the tectofugal pathways and a common downstream target of the PBGN, pulvinar, and VTA (Shang et al., 2015; Wei et al., 2015; Zhou et al., 2019). Considering the functional complexity and large volume of the amygdala, it was divided into the centromedial (CMA) and basolateral (BLA) nuclei based on an anatomical atlas (Amunts et al., 2005; Eickhoff et al., 2005), and the ROIs were further refined using functional connectivity with the superficial SC (Fig. 3A; see Section 2 for details). In the attended condition (Fig. 3B), the CMA showed a significant main effect of trajectory (F(2, 84) = 6.17, p = 0.003). Follow-up analysis revealed that this effect was driven by a significant trajectory effect in the upper visual field (F(2, 42) = 5.12, p = 0.01), with greater responses to hit-nasion than to near-miss trajectories (t(22) = 3.2, p = 0.008, d = 0.799). No significant effects of trajectory or related interactions were observed in the BLA (all p > 0.496). When ROIs were defined directly from the atlas, results were similar although less significant (CMA: ATT-UVF: trajectory effect: p=0.048, hit-nasion vs. near-miss: p = 0.032; BLA: ATT-UVF: trajectory effect: p=0.721, hit-nasion vs. near-miss: p = 0.5), likely due to the inclusion of more functionally irrelevant voxels.

To summarize, the results showed that top-down attention selectively enhanced collision sensitivity (hit-nasion vs. near-miss) in the superficial SC, PBGN, and CMA, particularly for looming stimuli in the upper visual field.

### 3.2 Minimal collision sensitivity in the geniculostriate pathway and higher cortex

To determine whether the collision sensitivity observed in the SC, PBGN, and CMA originated from the geniculostriate pathway and higher cortical regions, we examined fMRI responses to looming stimuli in the lateral geniculate nucleus (LGN) of the thalamus, early visual cortices, high-order motion sensitive areas (Field et al., 2020; Hervais-Adelman et al., 2015; Tyll et al., 2013), and frontoparietal regions defined by the HCP-MMP1 atlas (Glasser et al., 2016). No significant effects of trajectory were found in these brain regions (Supplementary Figs. S5 and S6). To further quantify collision sensitivity, we calculated a collision-sensitive response (CSR), defined as

$$CSR=\frac{\beta_{hit\;nasion}+\beta_{hit\;eye}}{2}-\beta_{near\;miss}$$

where β represents the fMRI response in one condition. No significant CSR was observed in the ROIs of the early visual areas or other cortical areas in a whole-brain analysis (Fig. 4). Using hit nasion vs. near miss as the collision-sensitive response yielded similar results. These findings suggest that the collision sensitivity observed in the subcortical areas is unlikely to have originated from the geniculate-striate pathway or higher cortical regions.

**Fig. 4.**
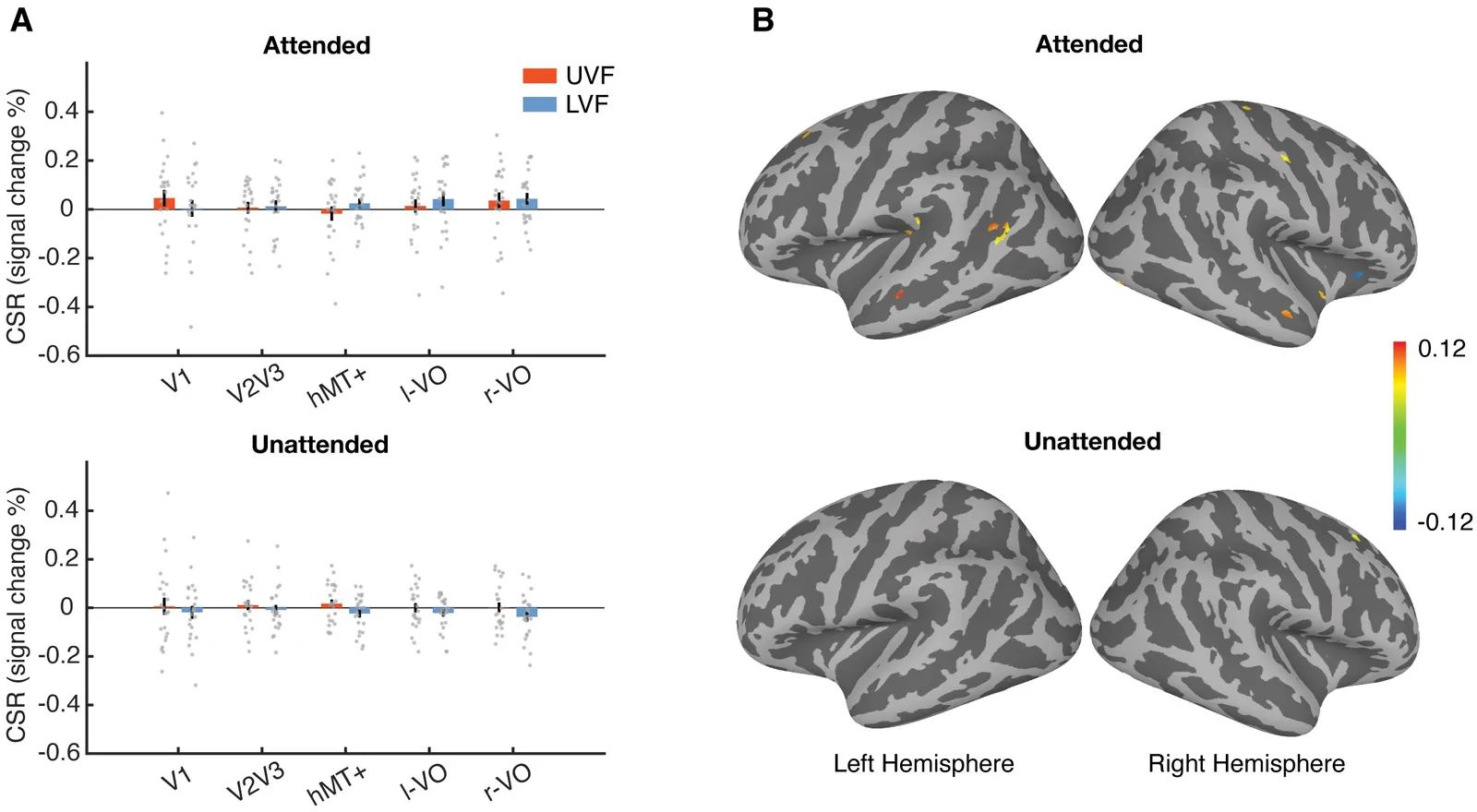
Collision sensitive response (CSR) in cortical areas. (A) CSR, defined as response difference between the collision (hit-nasion and hit-eye) and near-miss trajectories, in early visual areas. (B) CSR in other cortical regions. The color bar indicates CSR in percent signal change. Maps were presented with a threshold of uncorrected p < 0.001. No clusters survived following FWE correction. The results were comparable when the responses in the upper and lower visual fields were combined or when they were analyzed separately (data not shown). For retinotopic regions including V1, V2/V3, and hMT+, responses to contralateral looming stimuli in ROIs of both hemispheres were averaged. For VO and whole-brain analysis, responses to looming stimuli in the contralateral and ipsilateral visual fields were averaged. hMT+, human middle temporal complex; l/r-VO, left/right ventral occipitotemporal cortex. Other conventions as in Figure 2.

### 3.3 Different subcortical pathways exhibit collision sensitivity under attended and unaware conditions

To investigate information flow in the subcortical pathways underlying collision detection, we employed correlation and path analysis of collision-sensitive responses (CSRs), along with beta series connectivity analysis (Cisler et al., 2014; Rissman et al., 2004). We first examined pairwise correlations between CSRs. A significant correlation was found between CSRs in the superficial SC and PBGN in the ATT-UVF condition (r = 0.446, p = 0.033, Fig. 5A), but not in the other conditions (all r < 0.2). To investigate directed information flow, we constructed a structural equation model (SEM) using the across-subject correlations of CSRs between brain regions (Jun et al., 2020; Zhuang et al., 2005). The model (Fig. 5B) included connections among the superficial SC, PBGN, pulvinar, VTA, and CMA were defined by the anatomical projections in primates (May, 2006; Rafal et al., 2015; Zubair et al., 2021) and established neural circuits in rodents (Shang et al., 2015; Wei et al., 2015; Zhou et al., 2019). For each attention × visual field condition, we fitted the SEM to the ROI covariance matrix to estimate path-specific connectivity strengths (Fig. 5C; Supplementary Fig. S7). In the ATT-UVF condition, this analysis revealed significant connectivity for the SC-PBGN (β = 0.45, p = 0.017) and SC-VTA (β = 0.41, p = 0.029) pathways. Critically, the SC-PBGN connection was significantly stronger in the attended than in the unattended condition for stimuli presented in the UVF (p < 0.001). Consistent with the path analysis, beta series functional connectivity of collision responses revealed the strongest coupling between SC and PBGN for stimuli presented in the UVF during the attended condition (F(2, 44) = 4.51, p = 0.017; SC-PBGN vs. SC-Pul: p = 0.014, Fig. 5E ATT-UVF).

**Fig. 5.**
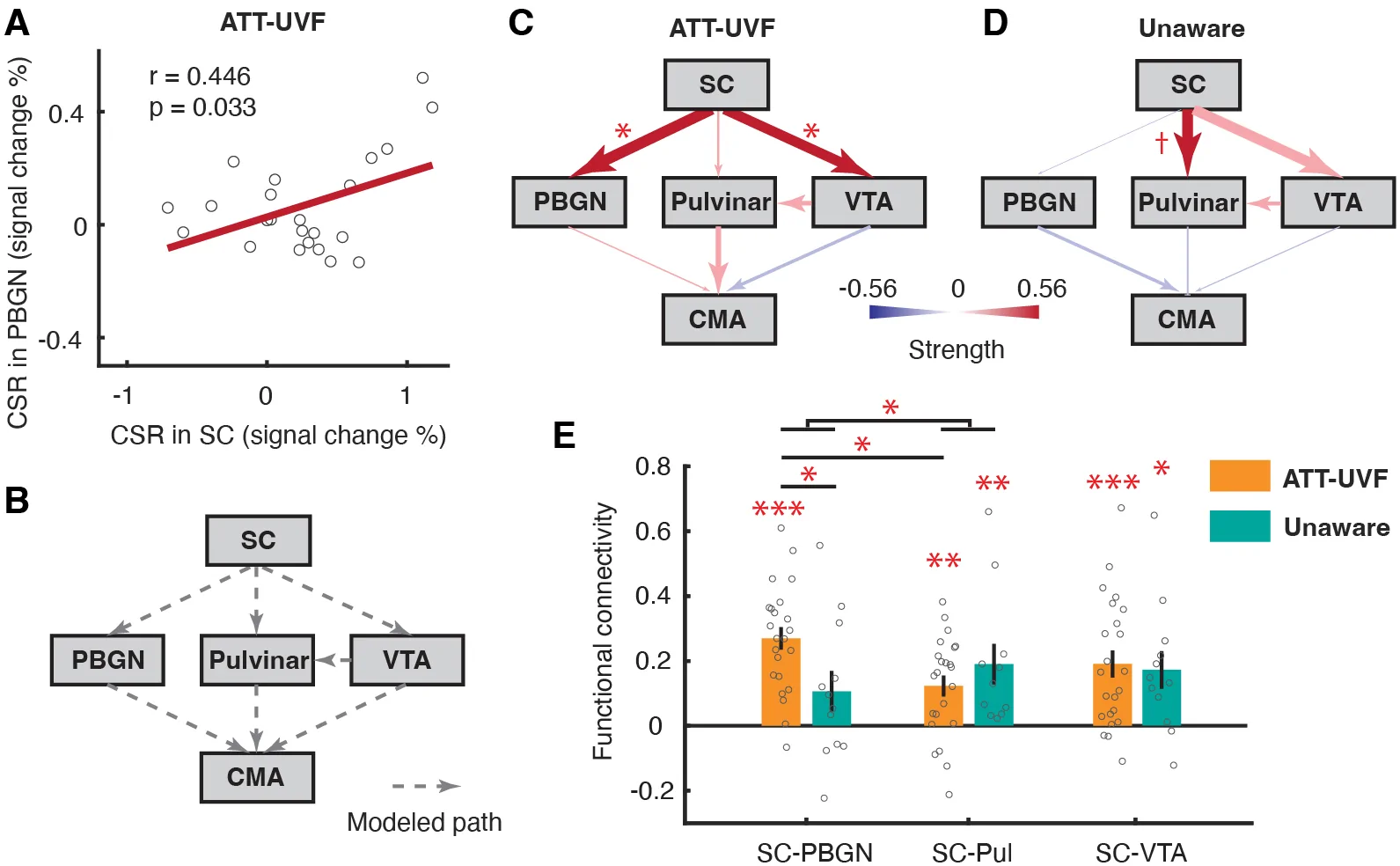
Correlation and connectivity analyses in the subcortical regions. (A) Scatterplot of collision sensitive responses (CSRs) in the PBGN and the superficial SC in the ATT-UVF condition. Red line indicates linear fit of individual data (gray circles). (B) The structural equation model (SEM) for the path analysis. Gray dashed arrows indicate connections based on known anatomical projections. (C) SEM results in the ATT-UVF condition in healthy participants. (D) SEM results for looming stimuli presented in the blind visual field of hemianopic patients. Dark and light colors depict significant and non-significant connections, respectively. (E) Functional connectivity assessed using beta series methods for the hit trajectories. ATT-UVF and Unaware represent healthy and patient data, respectively. ***p < 0.001; **p < 0.01; *p < 0.05; ^†^p < 0.1.

It has been proposed that subcortical pathways can process important visual information in the absence of visual awareness(Kinoshita et al., 2019; Pasley et al., 2004; Schmid et al., 2010). In support of this, our previous study showed that SC and its downstream subcortical nuclei, including VTA and pulvinar, are sensitive to collision trajectory even without awareness and attention to the looming stimuli in hemianopic patients with unilateral lesions of the geniculostriate pathway (Guo et al., 2024). To further investigate how top-down attention and awareness influence the functional connectivity of these subcortical pathways, we re-analyzed the patient data from our previous study (Guo et al., 2024). Visual stimuli were similar to the hit-eye and near-miss conditions used in the current study, except that they were presented on a 2D screen. For collision-sensitive responses to looming stimuli presented in the blind visual field, the path analysis shows no significant connection (only insignificant trend for the SC-pulvinar connection: β = 0.44, p = 0.095; Fig. 5D Unaware). However, beta series connectivity analysis revealed significant SC-pulvinar (p = 0.004, Fig. 5E Unaware) and SC-VTA (p = 0.015) connections, whereas the SC-PBGN connection was not significant (p = 0.125).

Thus far, our results suggest that SC-PBGN and SC-pulvinar pathways are involved in collision detection in attended (healthy participants) and unaware (hemianopic patients) conditions, respectively. To further confirm these differences, we performed LME analysis on the functional connectivity data. The results showed a significant interaction (F(1, 65) = 6.30, p = 0.015) between the pathways (SC-PBGN / SC-Pulvinar) and groups (healthy / patients). Follow-up tests revealed a significant difference in SC-PBGN connectivity between the two groups (p = 0.018; Fig. 5E).

## 4 Discussion

In the present study, we used fMRI to investigate collision sensitivity to looming stimuli in human subcortical pathways originating in the superior colliculus (SC). Superficial SC, PBGN, and centromedial amygdala (CMA), exhibited sensitivity to collision trajectories under top-down attention, but not when attention was diverted away from the looming stimuli by a demanding RSVP task at central fixation. Collision sensitivity was strongest for hit-nasion trajectories presented in the upper visual field, consistent with the behavioral results. Correlation and path analysis of collision sensitivity and beta series functional connectivity consistently indicate that the SC-PBGN pathway is involved in collision detection in healthy participants during the attended condition, whereas the SC-pulvinar and SC-VTA pathways are involved in collision detection without awareness to stimuli presented in the blind visual field of hemianopic patients.

Neural circuits formed by parvalbumin-positive (PV+) excitatory neurons in the superficial SC, along with their downstream targets in the PBGN and amygdala, have been shown to mediate defensive responses to looming stimuli in rodents (Shang et al., 2015, 2018). The current study further reveals that the SC-PBGN pathway processes collision trajectory information in humans, but only when participants are paying attention to the looming stimuli. Given the ~1.5-mm thickness of the superficial and the intermediate layers of the SC (Tardif & Clarke, 2002) and the 2-mm fMRI resolution, ROIs for the superficial SC may also include responses from the intermediate layers. However, collision-sensitive activations were significant in the retinotopic regions in the superficial SC (Katyal et al., 2010; Schneider & Kastner, 2005) but not in the intermediate SC, suggesting that top-down attention preferentially modulates activity of visuosensory neurons in the superficial SC for detecting collision trajectories. This is aligned with an important role of the SC in the control of attention and eye movements (Katyal & Ress, 2014; Katyal et al., 2010; Lovejoy & Krauzlis, 2017; Schneider & Kastner, 2009): top-down priority may interact with bottom-up saliency to guide attention and eye movements toward threatening visual events. The observed collision sensitivity in fMRI responses was unlikely caused by fixational eye movements because observers could maintain stable fixations with no significant difference between hit and miss conditions, as demonstrated in an additional eye-tracking session outside the scanner (Supplementary Fig. S8).

Collision-sensitive responses were stronger for the hit-nasion than the hit-eye trajectory, implying that binocular information is processed by the subcortical pathway, likely contributing to accurate estimation of collision trajectories. In support of this explanation, neurons in the primate SC receive binocular retinal inputs (Dilbeck et al., 2022), and some tuned to opposite motion directions from the two eyes (Tailby et al., 2012), consistent with the retinal motion produced by the hit-nasion trajectory. A binocular region in the rodent SC connected with the PBGN also facilitates detecting looming stimuli in the UVF (Deichler et al., 2020). Thus, it is possible that top-down attention may selectively enhance the sensitivity of binocular neurons in the SC-PBGN pathway to improve the accuracy of collision detection. The connectivity analyses suggest that the SC-VTA pathway may also be associated with collision detection in the attended condition in healthy participants (Fig. 5C, 5E). The absence of collision-sensitive responses in the VTA itself may reflect its high sensitivity to all looming stimuli, regardless of trajectory (Fig. 3B). While activity in the LGN and frontoparietal attention networks did not differ significantly between collision and near-miss trajectories, strong attentional modulation of BOLD responses to looming stimuli was observed in these regions (Supplementary Figs. S5 and S6).

Our previous study found stronger SC responses to near-miss trajectories in the UVF than those in the LVF (Guo et al., 2024), whereas no significant difference was observed for collision trajectories, likely due to response saturation at a short TTC (33 ms). Using looming stimuli at a longer TTC (138 ms), the current study revealed a clear UVF advantage in subcortical responses to collision compared to near-miss trajectories, aligned with behavioral findings of higher collision sensitivity in the UVF than in the LVF (Fig. 1). Consistent with our human data, the tectofugal pathways in rodents also mediate defensive responses particularly to looming stimuli in the UVF (Deichler et al., 2020; Isa et al., 2020; Wei et al., 2015; Zhou et al., 2019). Moreover, the SC in non-human primates also overrepresents the UVF with sharper, stronger and faster neural responses (Hafed & Chen, 2016). This UVF advantage for detecting approaching objects from a distance stands in contrast with the pervasive LVF advantage observed in human behaviors within peripersonal space (Himmelberg et al., 2023). This functional dissociation may reflect the adaptive specialization of distinct neural pathways (Carrasco et al., 2022; Tuncok et al., 2025). Cortical pathways, shaped by the demands of bipedal terrestrial life, exhibit a preferential bias for visual processing in the LVF to facilitate manual tasks and ground navigation (Himmelberg et al., 2022, 2023), whereas subcortical pathways are phylogenetically conserved and ecologically adapted to detect threats from the UVF, such as aerial predators. They further support the idea that the superficial layers of the human SC are sensitive to the time-to-collision of looming stimuli, consistent with the findings in birds (Wu et al., 2005) and rodents (Shang et al., 2018).

During passive viewing, previous fMRI studies have demonstrated that the SC responds more strongly to looming than to receding stimuli (Cléry et al., 2020; Thieu et al., 2024, 2026). Our previous study (Guo et al., 2024) and the new functional connectivity analysis (Fig. 5F) suggest that the SC-pulvinar and SC-VTA pathways are involved in the automatic detection of collision trajectories even without awareness and attention to looming stimuli in the blind visual field of hemianopic patients. These findings suggest that SC-pulvinar and SC-VTA pathways may serve as “low road” shortcuts for automatic processing threatening, emotional, and social information from exteroceptive input (Kragel et al., 2021; Pessoa & Adolphs, 2010). In contrast to the findings in hemianopic patients, we did not observe significant collision sensitivity in the SC and its downstream subcortical nuclei in the unattended condition for healthy participants. This is likely attributable to the attentionally demanding RSVP task at fixation, which may deplete the attentional resources required for collision detection in the SC. An alternative explanation is that, for hemianopic patients, post-lesional functional reorganization may strengthen subcortical collision-detection pathways (Isa & Yoshida, 2009; Tamietto et al., 2012).

In rodents, divergent tectofugal and thalamic pathways have been shown to mediate different types of defensive behaviors, such as escaping and freezing (Salay et al., 2018; Shang et al., 2018). Our data suggest that in humans, these tectofugal pathways are differently engaged in collision detection depending on the state of attention and consciousness: the SC-PBGN pathway, under top-down attention, shows response patterns related to the precise estimation of collision trajectories with potential binocular integration, whereas the SC-pulvinar and SC-VTA pathways are associated with collision detection without conscious awareness.

## Supplementary Material

Supplementary Material

## Data Availability

Data and code to reproduce the main findings of this study are publicly available at Zenodo.org (https://doi.org/10.5281/zenodo.21292233).
