# Supplementary material for: Attention selectively enhances collision sensitivity in the superficial SC, PBGN and centromedial amygdala to looming stimuli in the upper visual field
Source: Imaging Neurosci (Camb). 2026 Oct 29;4:IMAG.a.1432. doi: 10.1162/IMAG.a.1432 (PMC13722050; doi:10.1162/IMAG.a.1432)
Supplement: Supplementary file 1 [file IMAG.a.1432_supp.pdf]

SI figures

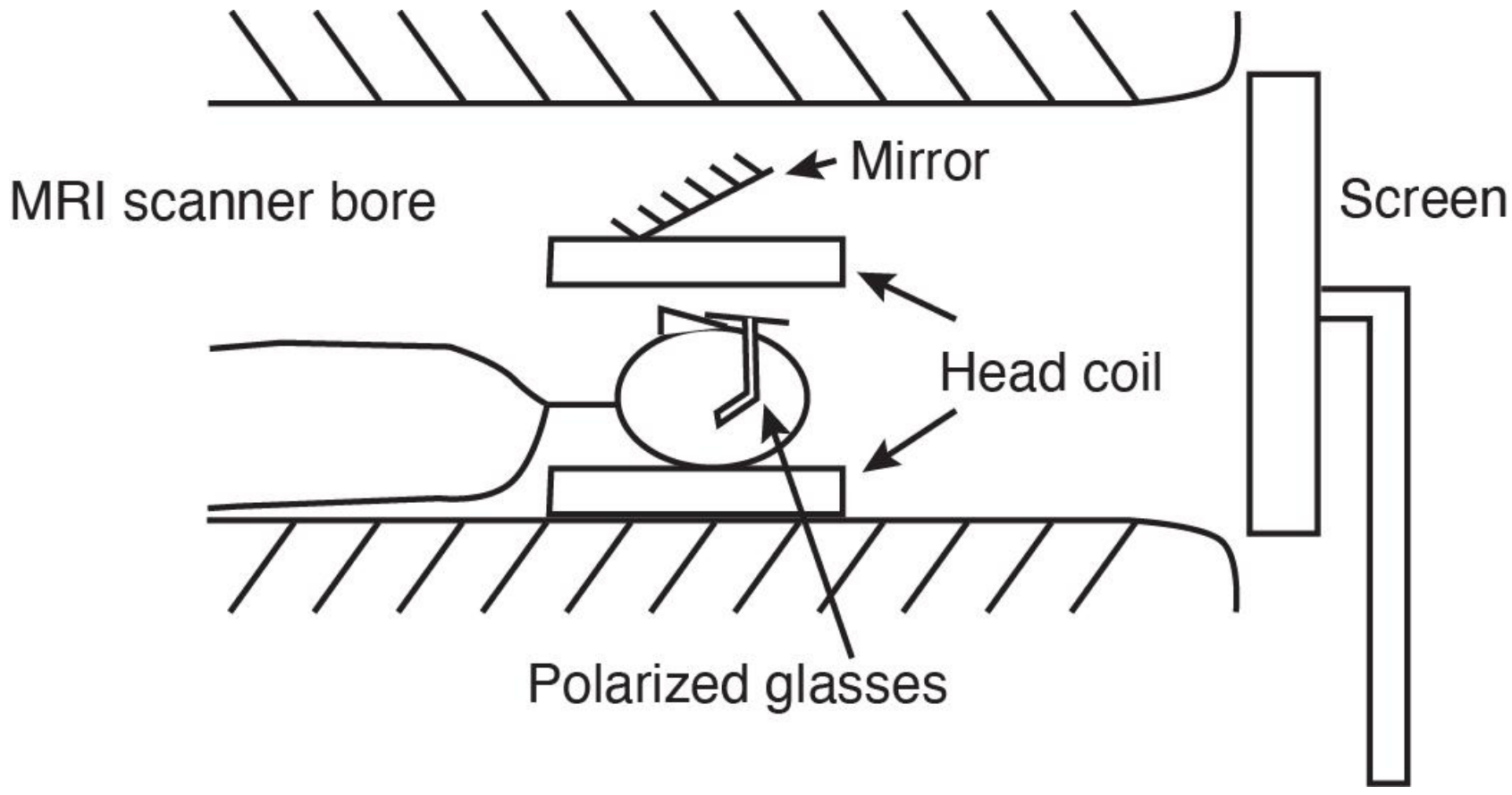

Fig. S1. Schematic diagram of the experiment setup for stimulus presentation. In the experiment, the screen was placed at the head end of the scanner bore. Participants viewed the screen through a mirror mounted on the head coil, and wore polarized 3D glasses to enable stereoscopic perception of the stimuli.

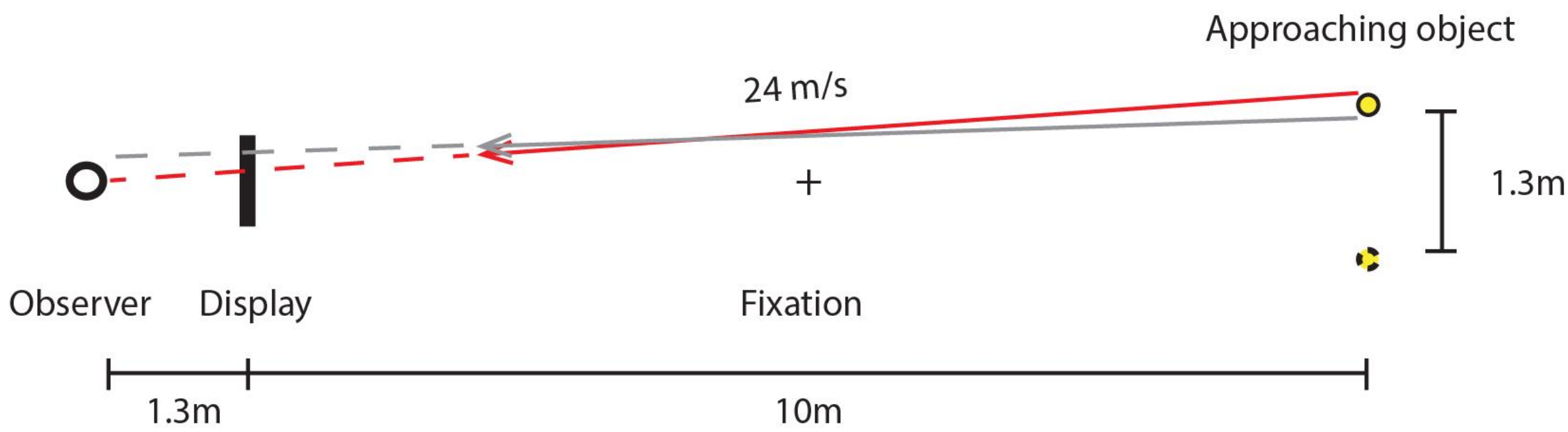

Fig. S2. Top view of the stimulus trajectories. This schematic is a scaled-down version of the scenes simulated in the experiment. Only hit-nasion (red) and near-miss (gray) trajectories were plotted. The point of collision and passage were indicated by the extended dashed lines.

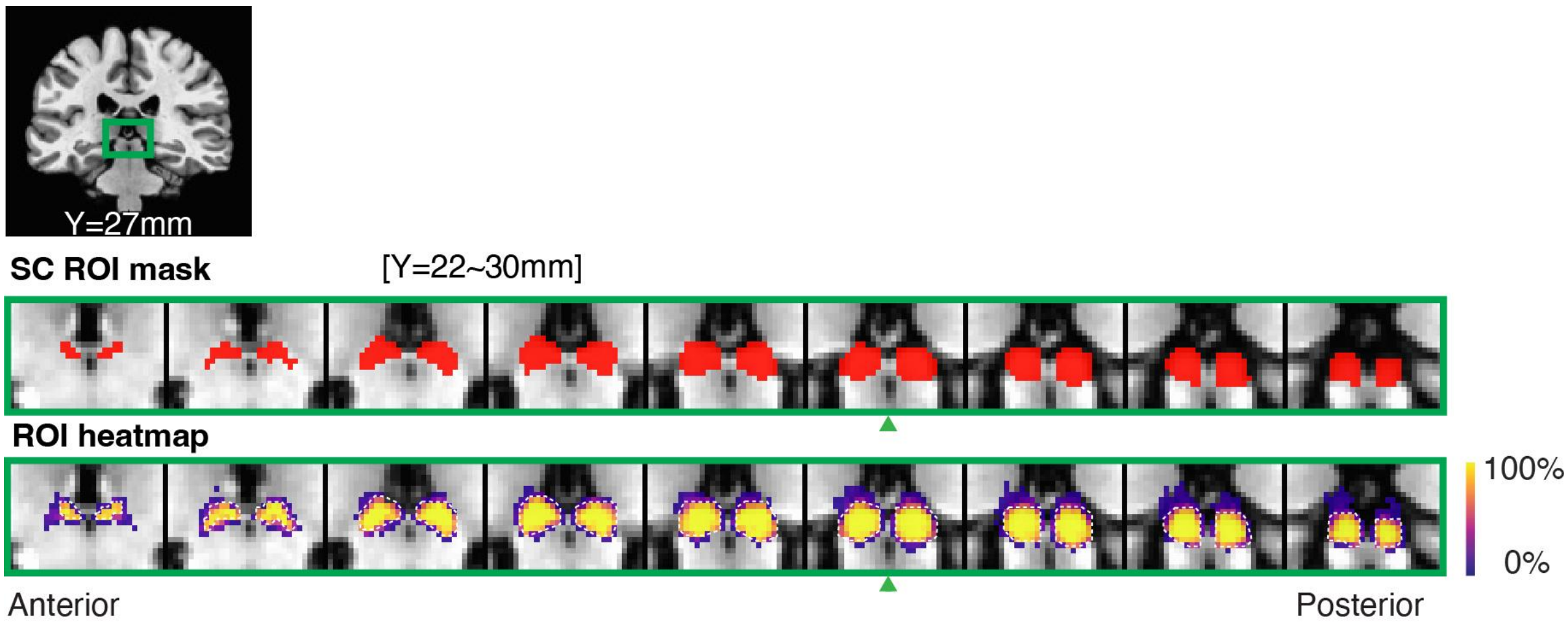

Fig. S3. The ROI definition of the SC. The red color in the upper row represents the ROI mask defined in the template space (AFNI TLRC). To demonstrate the quality of nonlinear spatial normalization of subcortical areas, the ROIs of the SC were also carefully defined in the native space, then transformed to the template space, and used to generate the ROI heatmap in the bottom row. The color bar indicates the probability of voxel-wise overlap across all subjects. The heatmap reveals a highly consistent spatial location across subjects in the template space after normalization. White dashed lines represent the SC boundaries in the template space. Coronal slices were presented from anterior to posterior at a 1-mm step. The enlarged slice corresponding to the full image is marked by a triangle below. Coordinates are in AFNI TLRC space (positive Y = posterior).

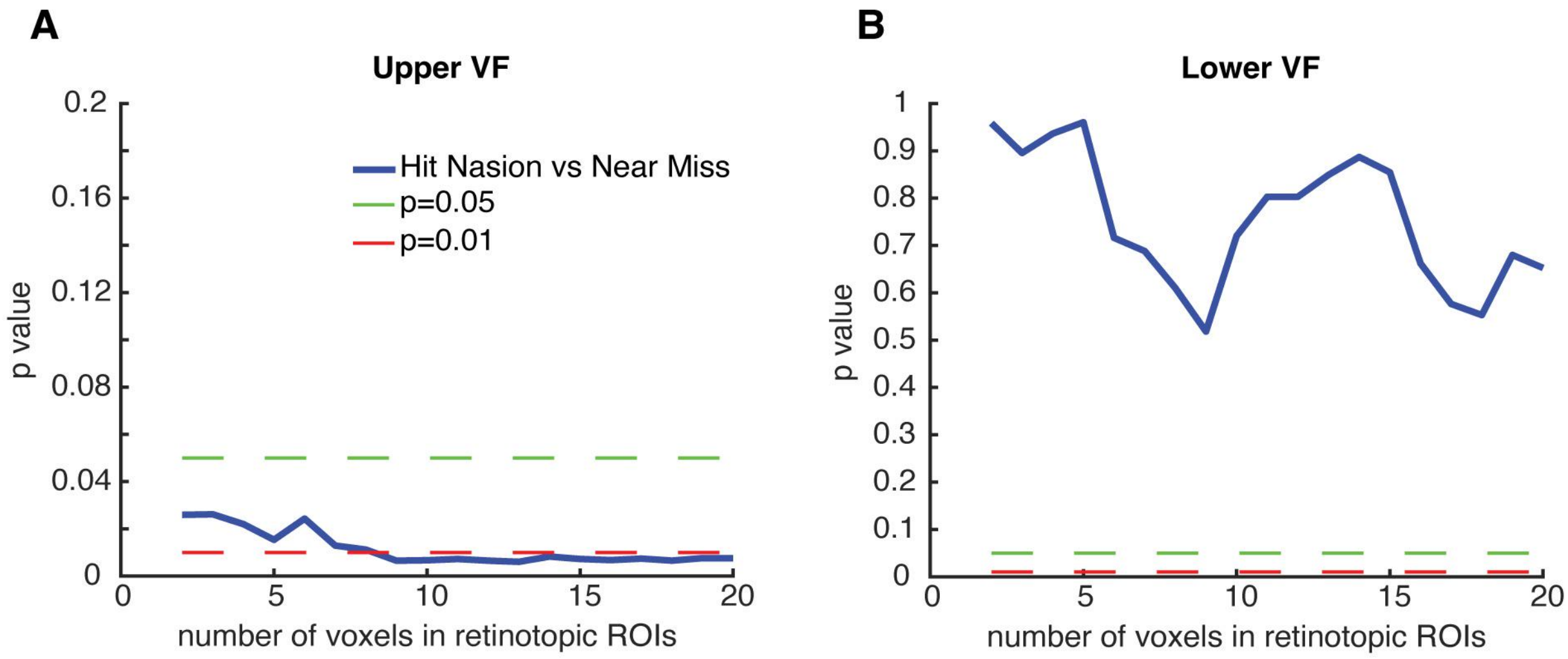

Fig. S4. Statistical significance (p-values) of collision sensitivity as a function of voxel number in the retinotopic ROIs of the superficial SC. The number of voxels in the retinotopic ROIs was controlled by the threshold of contralateral vs. ipsilateral activation in the localizer. Collision sensitivity (hit nasion vs. near miss in the attended conditions) remained significant in the upper (A) visual field, and insignificant in the lower (B) visual field at different ROI-selection thresholds.

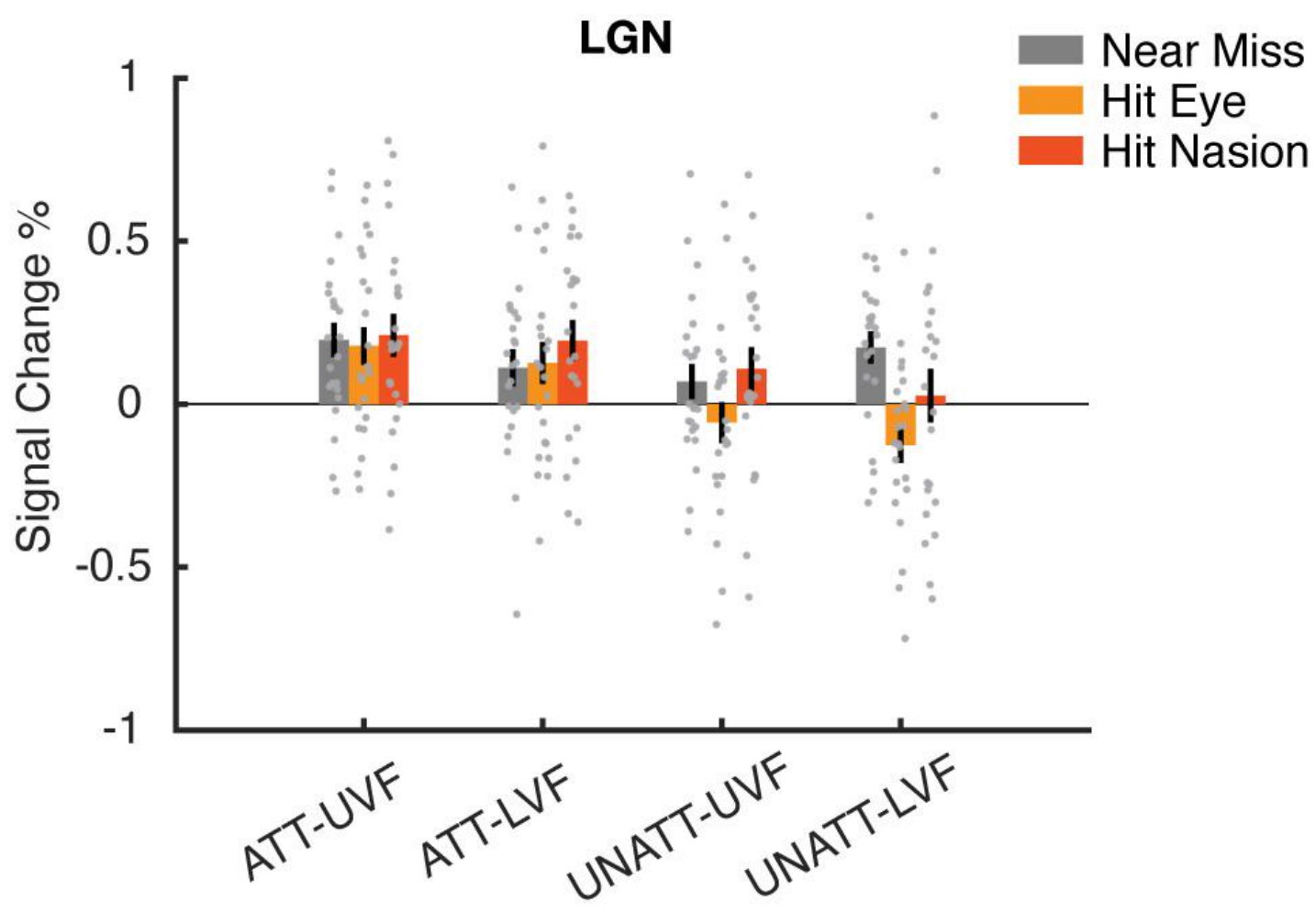

Fig. S5. Looming responses in the LGN. Dots denote the data of individual participants. Error bars indicate S.E.M. ATT, attended; UNATT, unattended; UVF, upper visual filed; LVF, lower visual field.

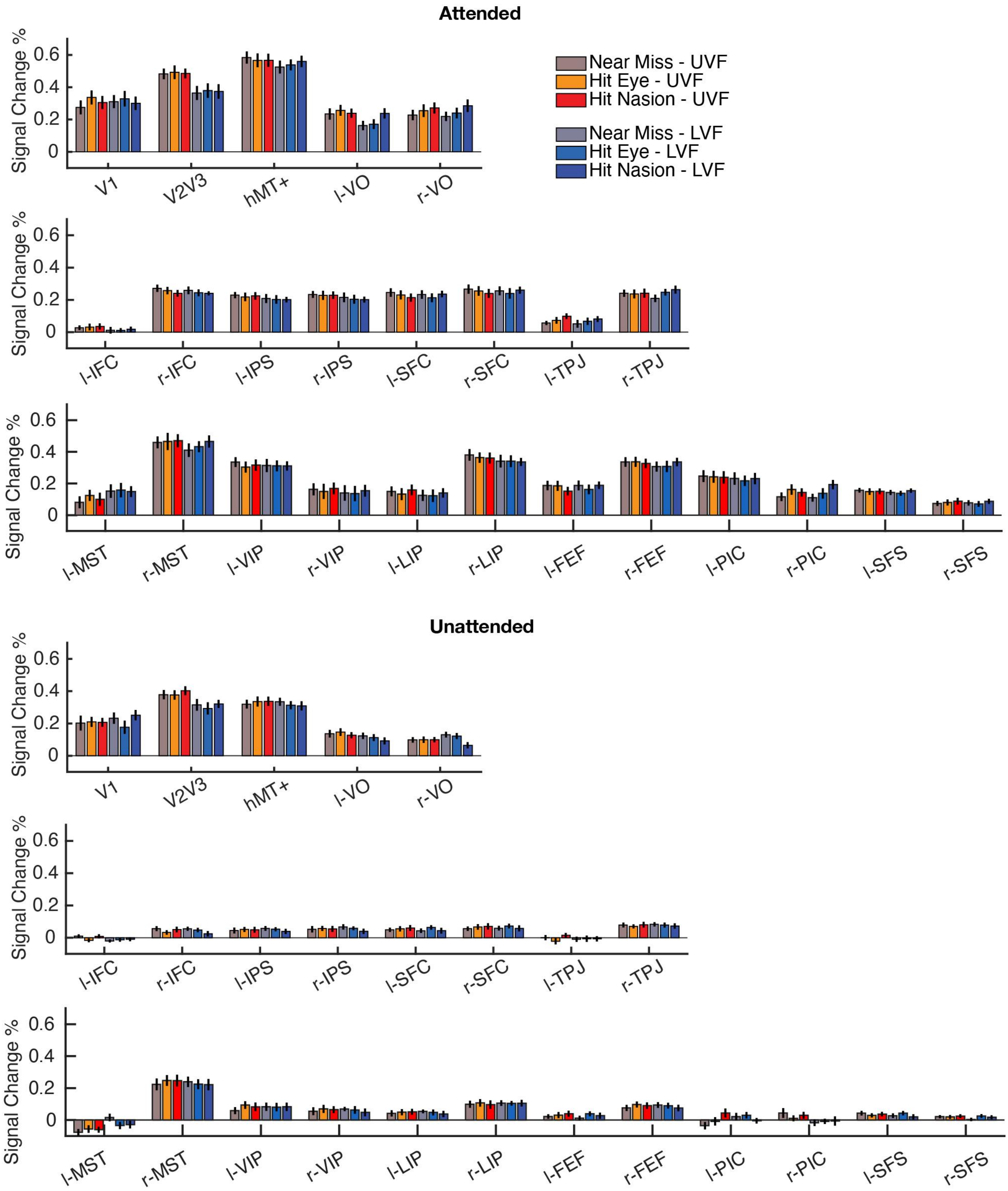

Fig. S6. Looming responses in the visual cortices, frontoparietal attention networks and higher-level motion-sensitive areas. For V1, V2/V3, and hMT+, results were obtained from bilateral ROI responses to contralateral stimuli. For other cortical areas, responses from the left and right hemifields were averaged. We performed a LME

analysis for each ROI, separately for the attended and unattended conditions and for the UVF and LVF conditions. No significant effect of trajectory was found (all uncorrected $p > 0.05$). UVF, upper visual filed; LVF, lower visual field. hMT+, human middle temporal complex; VO, ventral occipital cortex; IFC, inferior frontal cortex; IPS, intraparietal sulcus; SFC, superior frontal cortex; TPJ, temporoparietal junction; MST, medial superior temporal area; VIP, ventral intraparietal area; LIP, lateral intraparietal area; FEF, frontal eye field; PIC, posterior insular cortex; SFS, superior frontal sulcus. l/r-, left/right hemisphere.

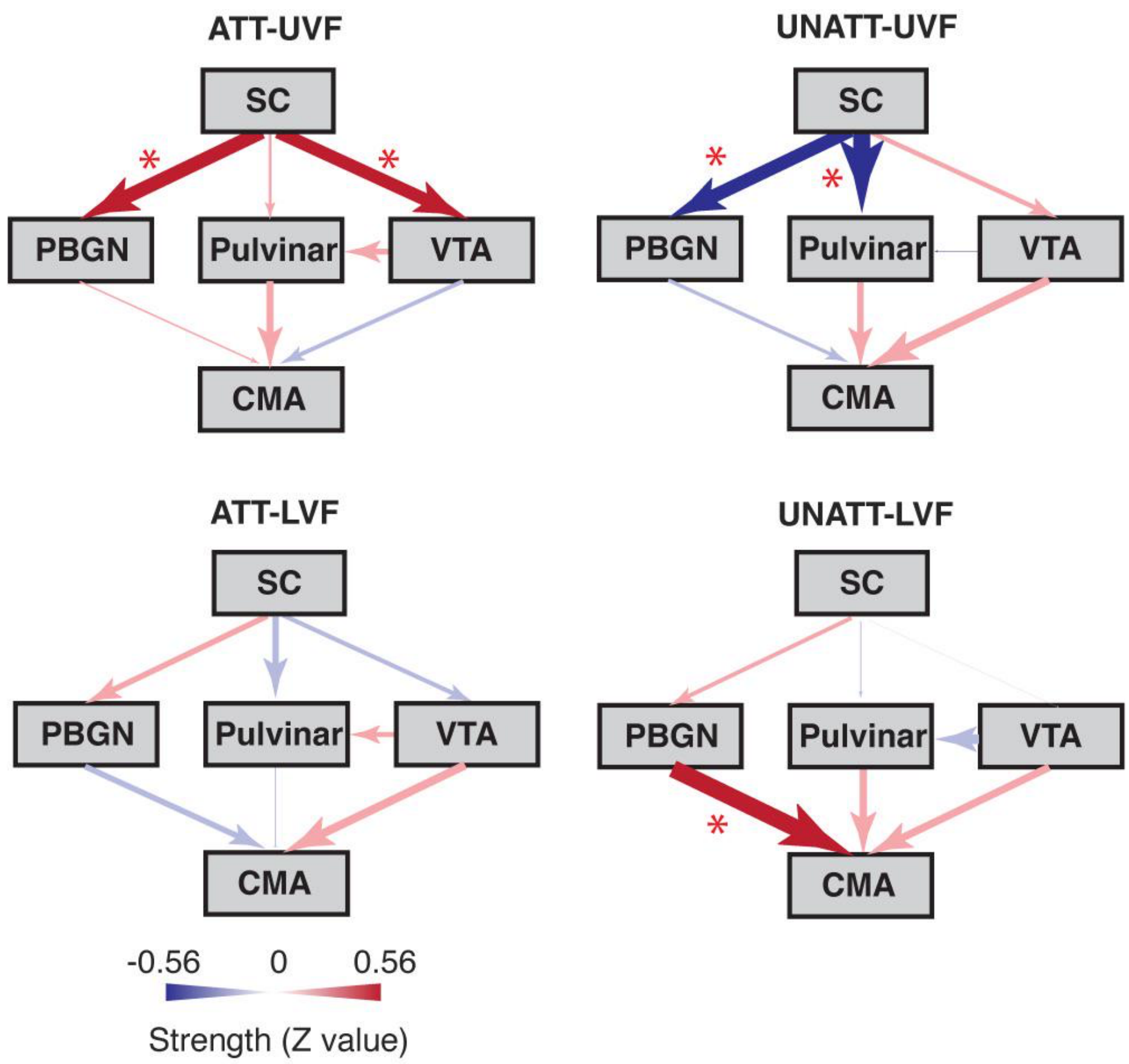

Fig. S7. Path analysis results in all stimulus conditions. Dark and light colors depict significant and non-significant connections, respectively. ATT, attended; UNATT, unattended; UVF, upper visual filed; LVF, lower visual field. * p<0.05.

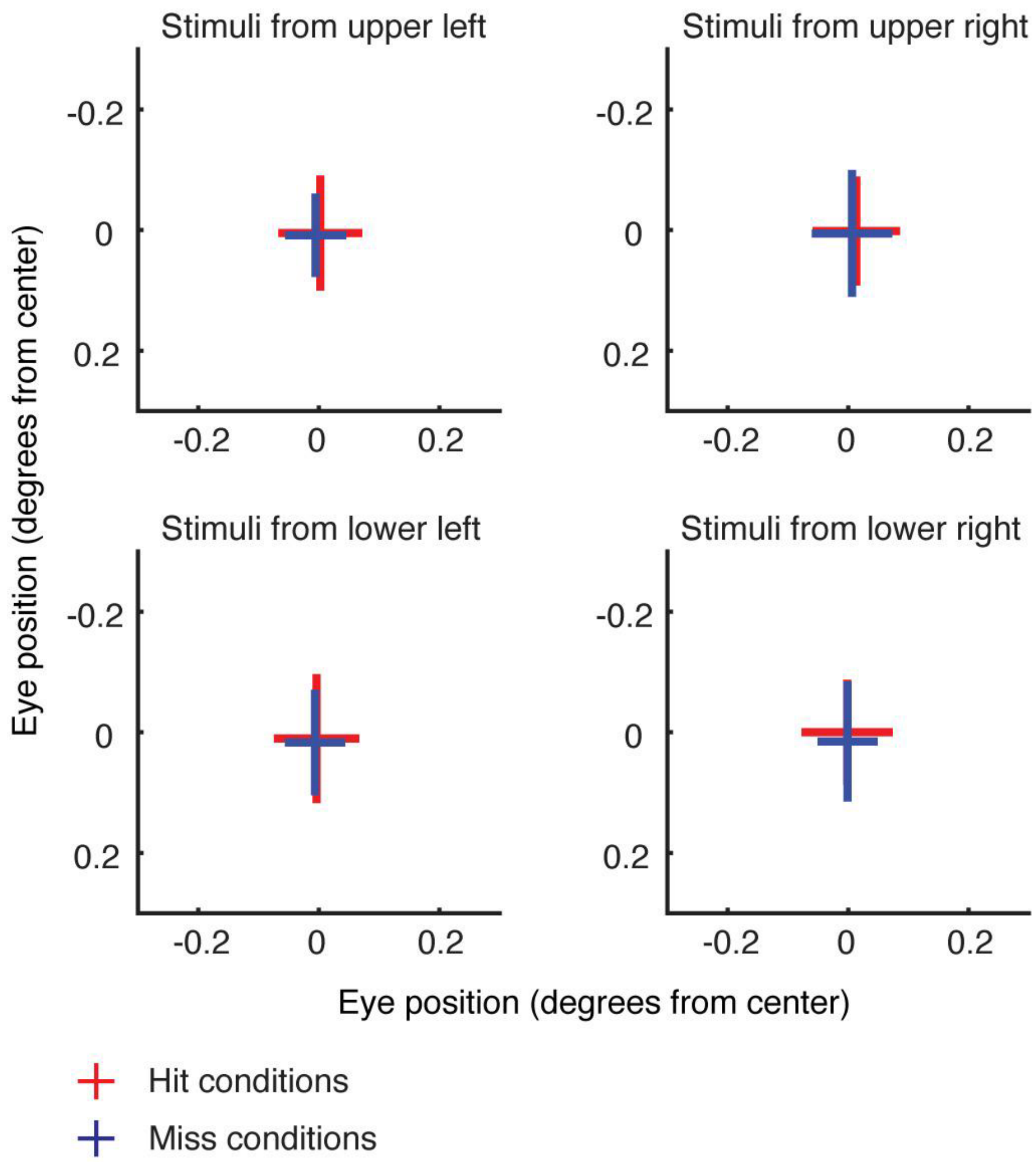

Fig. S8. Eye gaze positions for looming stimuli from four quadrants of the visual field. During the eye-tracking session, eye gaze data were analyzed from stimulus onset to 1,000 ms afterward. Error bars indicate three standard deviations across subjects. No significant differences were found between hit (hit nasion + hit eye) and miss (near miss + far miss) trajectories or between stimuli from different quadrants of the visual field. Far miss: the trajectory was determined to result in approximately 2% hit responses (impact point: 9.5 cm from the nasion on average).
